# Social and economic problems of ICU survivors identified by a structured social welfare consultation

**DOI:** 10.1186/s13054-019-2442-5

**Published:** 2019-05-02

**Authors:** J. M. McPeake, P. Henderson, G. Darroch, T. J. Iwashyna, P. MacTavish, C. Robinson, T. Quasim

**Affiliations:** 1 Glasgow Royal Infirmary, ICU, NHS Greater Glasgow and Clyde, Glasgow, G31 2ER UK; 2 School of Medicine, Dentistry and Nursing, University of Glasgow, Glasgow, UK; 3 Center for Clinical Management Research, VA Ann Arbor Health System, Ann Arbor, MI USA; 4 Department of Internal Medicine, Division of Pulmonary and Critical Care, University of Michigan, Ann Arbor, MI, USA

**Keywords:** Rehabilitation, Critical care, Recovery, Post intensive care syndrome, Long term outcomes

Despite over a decade of trials, no outpatient intervention has demonstrated any measurable improvement in the poor health-related quality of life (HRQoL) patients experience following critical illness [1, 2]. One novel avenue is to alleviate the socio-economic impact of critical illness. These are important in isolation, but also crucial mediators of patient depression, anxiety, and HRQoL [3, 4].

To identify opportunities for further innovation, we identified the socio-economic support patients and caregivers sought during the recovery period.

Intensive Care Syndrome: Promoting Independence and/or Return to Employment (InS:PIRE) is a five week rehabilitation programme for intensive care unit (ICU) survivors and their caregivers [5]. During this multi-disciplinary intervention, a social welfare consultation is available to participants. This one-to-one consultation offers patients and caregivers the opportunity to seek advice about any socio-economic problems they are experiencing following critical illness. Data for this letter was generated from an ongoing service evaluation, over a 20-month period in a single site in the UK. NHS Greater Glasgow and Clyde Caldicott Guardian approved this study

Thirty-one percent of patients (33 of 108 patients who attended) and two caregivers requested a consultation (Table 1). Approximately one fifth (*n* = 7) of patients required more than one appointment, and two individuals raised more than one issue. Thus, 42 patient and two caregiver issues were examined; these issues were classified under six categories**.**

**Table 1:** Patient demographics and financial and social advice sought

Patient demographic	Number (*n* = 33)
Gender, male (%)	18 (55%)
Age, years, median (IQR)	55 (50.5–68.5)
APACHE II* median (IQR)	20 (17–24.5)
Hospital Length of Stay Median (IQR)	37 (21–68)
Time between ICU discharge date and ICU follow-up appointment, days, median (IQR)	168 (132.5–244)
Issues discussed (patient)	Number (*n* = 42)
Welfare benefit advice	22 (52.5%)
Employment	7 (17%)
Adaptations and access to parking/mobility	4 (9.5%)
Debt/financial issues	4 (9.5%)
Housing	4 (9.5%)
Legal	1 (2%)

*Acute Physiology and Chronic Health Evaluation

Over 50% (*n* = 22) of issues raised concerned access to welfare benefits and allowances related to being out of work. Patients also needed information about returning to employment and the financial implications associated with this (*n* = 7, 17%). Other issues included housing, home adaptation, debt, and legal advice. Adaptations to housing included access to stairs and aids needed for basic care. On one occasion, debt advice was related to utility bills generated during hospitalisation. Patients also wanted support in relation to improving activities of daily living, for example, access to parking and mobility support (Table 1).

The two caregivers sought information on housing adaptations and benefits. Both caregiver issues required follow-up from community services as did 38% (*n* = 16) of patient issues. The remaining issues were resolved during the consultation or through information provision.

This work demonstrates that delivering socio-economic support during ICU rehabilitation is feasible and that the social-economic needs of this group are diverse. A larger sample is required to understand if these findings are similar internationally. This information should be utilised to refine future research in this area.

## Abbreviations

HRQoL: Health-related quality of life
ICU: Intensive care unit
InS:PIRE: Intensive Care Syndrome: Promoting Independence and Return to Employment
